# Predicting Variation of Folk Songs: A Corpus Analysis Study on the Memorability of Melodies

**DOI:** 10.3389/fpsyg.2017.00621

**Published:** 2017-04-25

**Authors:** Berit Janssen, John A. Burgoyne, Henkjan Honing

**Affiliations:** ^1^Meertens Institute, Royal Netherlands Academy of Arts and SciencesAmsterdam, Netherlands; ^2^Music Cognition Group, Institute for Logic, Language and Computation, University of AmsterdamAmsterdam, Netherlands

**Keywords:** music information retrieval, music cognition, recall, memorability, stability, folk songs, corpus analysis

## Abstract

We present a hypothesis-driven study on the variation of melody phrases in a collection of Dutch folk songs. We investigate the variation of phrases within the folk songs through a pattern matching method which detects occurrences of these phrases within folk song variants, and ask the question: do the phrases which show less variation have different properties than those which do? We hypothesize that theories on melody recall may predict variation, and as such, investigate phrase length, the position and number of repetitions of a given phrase in the melody in which it occurs, as well as expectancy and motif repetivity. We show that all of these predictors account for the observed variation to a moderate degree, and that, as hypothesized, those phrases vary less which are rather short, contain highly expected melodic material, occur relatively early in the melody, and contain small pitch intervals. A large portion of the variance is left unexplained by the current model, however, which leads us to a discussion of future approaches to study memorability of melodies.

## 1. Introduction

Songs and instrumental pieces in a musical tradition are subject to change: as they are adopted by a new generation of listeners and musicians, they evolve into something new while retaining some of their original characteristics. The current article investigates to what extent this change of melodies may be explained by hypotheses on the memorability of melodies.

To address this question, we investigate a corpus of folk songs collected in the second half of the twentieth century, in which we can identify groups of variants. The variants are results of real-life melody transmission, something which would be difficult to study in an experimental setting, but for which the present folk song collection possesses high ecological validity. In folk song research, there is a long-standing interest in those melodic segments which resist change during melody transmission. This resistance to change is also referred to as *stability* (Bronson, 1951).

According to models of cultural evolution, the relative frequency of cultural artifacts can be explained based on *drift* alone: certain phrases might have been copied more frequently than others purely based on chance, and the relative stability of a given phrase in a collection of folk songs would be random (Henrich and Boyd, 2002). We hypothesize, instead, that stability can be predicted through the memorability of melodies.

To quantify stability, or the amount of variation a folk song segment undergoes through oral transmission, we follow Bronson's notion that “there is probably no more objective test of stability than frequency of occurrence.” (Bronson, 1951, p. 51). We formalize the relative stability of a melodic segment as its frequency of occurrence across variants of the same folk song. We focus on melodic phrases from the folk songs and employ a novel pattern matching method to determine whether or not a match for a given phrase may be found in a given folk song variant, based on similarity measures tested in Music Information Retrieval, and evaluated on a subset of folk songs in previous work (Janssen et al., in press). We then test whether there is a statistical relationship between a given phrase's matches in variants, and the same phrase's memorability, i.e., properties which might facilitate its recall.

Part of our predictions for the memorability of melodies are drawn from serial recall experiments, which typically test how well participants in studies remember word lists—presented visually or auditorily—or purely visual or spatial cues. Based on this research, we can expect that the length of a phrase might influence its memorability: a phrase with many notes contains more items that need to be correctly reproduced, and will therefore be harder to remember than a phrase with few notes. This does not take into account effects of chunking, which might reduce the memory load of phrases with many notes (Miller, 1956). Recall experiments with lists of different lengths have shown that increasing the length of a memorized list decreases the proportion of correctly recalled items (Ward, 2002). Moreover, rehearsal in the form of phrase repetitions might play a role: a phrase that is repeated several times within a melody might be memorized more faithfully than a phrase that only occurs once in each verse. The repetition can be considered rehearsal, which has been shown to increase retention of items (Murdock and Metcalfe, 1978).

Besides, the position of a melodic phrase within a piece might influence its memorability: in serial recall experiments, these effects are known as *serial position effects* (Deese and Kaufman, 1957). When the start of lists is remembered better, this is considered a *primacy effect* (Murdock, 1962). When words were presented auditorily, Crowder and Morton (1969) found that the end of lists were remembered better, which might lead one to expect that melodies, also auditory in nature, exhibit a *recency effect*. However, in Rubin's (1977) experiments on long-term retention of well-known spoken word passages (the Preamble to the constitution of the United States, Psalm 23, and Hamlet's monolog from the eponymous Shakespeare play), words at the start of such a passage are recalled better than items in the middle or at the end. As this situation is maybe closest to singing a folk song from memory, we assume that phrases at the start of melodies may also be more stable. Of course, serial position effects may be caused by an individual's more frequent exposure to items early or late in a melody (Ward, 2002), in which case we would expect that rehearsal is more important than serial position to explain the stability of melodic segments.

Next to these general hypotheses on recall, we test hypotheses based on melody recall research. Firstly, a significant body of research links melody recall to expectancy. According to Kleeman (1985), only music which can be processed by listeners based on their musical expectations, will be selected for transmission in a musical tradition (p. 17). Supporting this, Schmuckler (1997) found a relationship between expectancy ratings and melody recall in an experimental study on folk song melodies. To this end, 16 participants were instructed to rate how well artificial variants of 14 folk songs confirmed their expectancy. The variants of the folk songs were generated by scrambling the notes at the end of each song, maintaining the rhythmical structure and the end note. Afterwards, participants had to identify the melodies they had encountered in the first part of the experiment, presented along with previously unheard melodies. The hit rates were positively correlated with the expectancy rating, indicating that those melodies which conform best to melodic expectations of listeners are also most reliably recalled.

An alternative prediction would be that it is actually more unexpected items which are easier remembered. This is corroborated by evidence from free recall, where items which are unusual are usually better remembered (von Restorff, 1933). For music, Müllensiefen and Halpern (2014) found that memorability of melodies was increased if they contained a large amount of unique motifs, i.e., melodic material which is unusual and therefore unexpected. This means that expectancy may influence variation of melodies in opposing ways, which we both adopt as hypotheses (see hypotheses 4a and 4b in the list of hypotheses below).

Different formalizations of melodic expectancy exist, among which the influential implication-realization theory by Narmour (1990) predicts that the direction and distance, or *pitch interval*, between two ensuing musical tones implies the direction and size of the next pitch interval. Schellenberg (1996) quantified the principles that Narmour defined, such that for a given implicative pitch interval, there is a measurable expectancy of which note is likely to ensue. He performed three listening experiments in which listeners rated how well the last note fulfilled their expectations after listening to excerpts from British and Chinese folk songs, and from atonal music, and reanalyzed data from Unyk and Carlsen (1987). His experiments showed that the quantified implication-realization principles were highly correlated with listeners' expectancies.

Schellenberg found that Narmour's model can be reduced to two factors, *pitch proximity* and *pitch reversal*, without significant loss in explanatory power (Schellenberg, 1997). Hence, Schellenberg's simplified model can be considered a quantification of expectancy, which may predict how well a given melody is retained in a musical tradition.

Inspired by an article by Meyer (1957), Conklin and Witten (1995) approach expectancy with information-theoretical measures: according to Meyer's theory, expectancies are generated by learned probabilities of given events. A listener expects musical events she has heard frequently before, and will be surprised by musical events she hears for the first time. Conklin and Witten assume that this learning, and hence expectancy, can be based on different musical aspects, such as pitches, pitch intervals or durations, among others. For this, they developed a predictive model based on various musical aspects, which they refer to as *viewpoints*.

Conklin and Witten's model applies Prediction by Partial Matching (Cleary and Witten, 1984) to a given note event, expressed by one or several viewpoints. Prediction by Partial Matching (PPM) is a statistical model that is trained on the frequencies of *n-grams*, or sequences of *n* symbols, in a collection of documents, and which can then be used to predict a symbol in an unseen document given its context. In music prediction, the symbols are musical notes, described by various viewpoints, e.g., pitch, duration, pitch interval, or accentuation. If the model encounters a note sequence it has not seen in the learning phase, it will backtrack to the next shorter note sequence which it did encounter, and use the frequency of the shorter sequence to predict the following note.

Pearce and Wiggins (2004) extended Conklin and Witten's model such that the length of the musical sequence, or the order of the *n-gram*, is variable. Pearce and Wiggins confirmed that statistical information as modeled by their system, dubbed IDyOM (Information Dynamics of Music)^1^, predicts listener's expectancy ratings from various listening experiments on folk songs, hymns and single intervals to a great extent (Pearce and Wiggins, 2012).

Some recent corpus studies of popular music have indicated that the presence of repeating motifs in a melody or phrase may enhance its memorability. As such, Müllensiefen and Halpern (2014) investigated a large number of musical features derived from music notation of 80 Western pop songs, to see which of them would best predict the memorability of 80 pop song excerpts. The memorability was determined in a recall experiment with 34 participants, who listened to 40 excerpts and later were presented with all of the excerpts, having to indicate whether they had heard the song before, and how pleasant they considered the excerpt in question. The researchers considered responses on the pleasantness to represent *implicit* memory for the music, through the mere exposure effect (Zajonc, 1980). Müllensiefen and Halpern's results indicate that a melody is more easily remembered explicitly if it consists of motifs which are repeated within the melody. For the implicit memory of melodies, however, it was found that motifs should not repeat too much.

Van Balen et al. (2015) measure the memorability of pop songs that participants are likely to have heard through radio and other media. They register this memorability through reaction times in a game. The goal of the game is to indicate whether or not the player recognizes a given song segment (cf. Burgoyne et al., 2013). If the player's response is fast, Van Balen and colleagues surmise that the song segment in question is very memorable, or catchy. They use a range of features to predict the memorability of the melodies, among which the features used by Müllensiefen and Halpern (2014).

One of Balen and colleagues' strongest predictors of memorability turned out to be motif repetivity, which is in line with Müllensiefen and Halpern's findings on explicit melody recall. As our study focusses on melodies which were explicitly remembered by their singers, rather than pleasantness ratings of these melodies, we therefore adopt the prediction that motif repetivity will increase a phrase's stability. Motif repetivity can also be seen as related to chunking, as repeating motifs would provide meaningful subdivisions within a phrase. Chunking has been shown to facilitate learning in various domains (Gobet et al., 2001).

Based on the above observations, in the current paper we investigate the following five hypotheses of how variation of folk songs may be predicted through theories on melody recall:

1. Shorter phrases show less variation.
2. Phrases which repeat within their source melody show less variation.
3. Phrases which occur early in their source melody show less variation.
4. A phrases' expectancy is related to its variation.
  - Phrases which contain highly expected melodic material show less variation.
  - Phrases which contain highly surprising melodic material show less variation.
5. Phrases composed of repeating motifs show less variation.

## 2. Materials and methods

Our research was carried out using the folk song corpus (FS) from the Meertens Tune Collections^2^. This corpus comprises 4,125 digitized transcriptions of monophonic songs, of which the largest part has been recorded in field work between 1950 and 1980 (Grijp, 2008). 1,245 transcriptions originate from song books of the nineteenth and twentieth century known to contain variants to the recorded songs.

The corpus has been categorized into *tune families*, or groups of variants, by domain experts (c.f. Volk and van Kranenburg, 2012), and we use these pre-defined groups to investigate stability between song variants. We compare variants from the same tune family. Each variant is considered to represent the variation imposed by a particular singer or song book editor to a given melody. Consequently, we analyze which phrases of the songs belonging to a tune family vary more, or vary less between different variants: if a phrase occurs in many variants, this means that this phrase is less subject to change, or more stable.

To this end, we separate the FS corpus into three sub-corpora: (1) a training corpus of 360 melodies for which annotations of phrase occurrences were available; (2) a test corpus of 1,695 melodies with tune families comprising at least five variants, but excluding tune families from the training corpus; (3) a background corpus of 1,000 melodies with tune families comprising very few variants. All melodies which could potentially be related to melodies from the test corpus—because they might be hitherto unrecognized variants of a tune family in the test corpus (tune family membership undefined), or because they were subtypes of a tune family in the test corpus—were excluded from the background corpus.

The training corpus was used to train the computational method to find phrase occurrences; the background corpus was used to train information theoretical models; the test corpus was used to test the relationship between variation of the folk song phrases and their hypothesized memorability.

### 2.1. Detecting phrase occurrences

To quantify the amount of variation, or stability of a given melodic phrase (the query phrase), we detect its occurrences in melodies belonging to the tune family from which it was taken (its source tune family): the more variants of the source tune family the query phrase occurs in, the higher the stability of the phrase.

We detect occurrences through pattern matching, or the computational comparison of the query phrase to all melodies in its source tune family. The extent to which any segment in a given melody resembles the query phrase can be detected through various similarity measures. Earlier research on the above-mentioned training corpus with phrase occurrence annotations has shown that a combined measure of the similarity measures city-block distance (Steinbeck, 1982), local alignment (Smith and Waterman, 1981) and structure induction (Meredith, 2006) reproduces human annotations of phrase occurrences best. The similarity measures, as well as the way in which they were combined, are described in the Supplementary Material.

Research on the training corpus also showed which similarity score should be used as a threshold to separate between relevant occurrences (i.e., detected matches which were also annotated as instances of the query phrase) and irrelevant occurrences (i.e., detected matches which were not annotated as instances of the query phrase) for each of the three measures (Janssen et al., in press). This optimal similarity threshold results in the best trade-off between missing as few relevant occurrences as possible, while producing as few as possible irrelevant occurrences.

Our previous research indicated that the combined measure produces errors in comparison to human annotators, i.e., it misses about 30% of the relevant occurrences, and detects about 8% irrelevant occurrences. The percentage of produced errors differs depending on the analyzed tune family. Using the pattern matching procedure, for the 9,639 phrases from 147 tune families in the test corpus, we receive 170,803 computational judgements on the occurrences of these phrases in their respective source tune families.

### 2.2. Formalizing hypotheses

**Figure 1:**
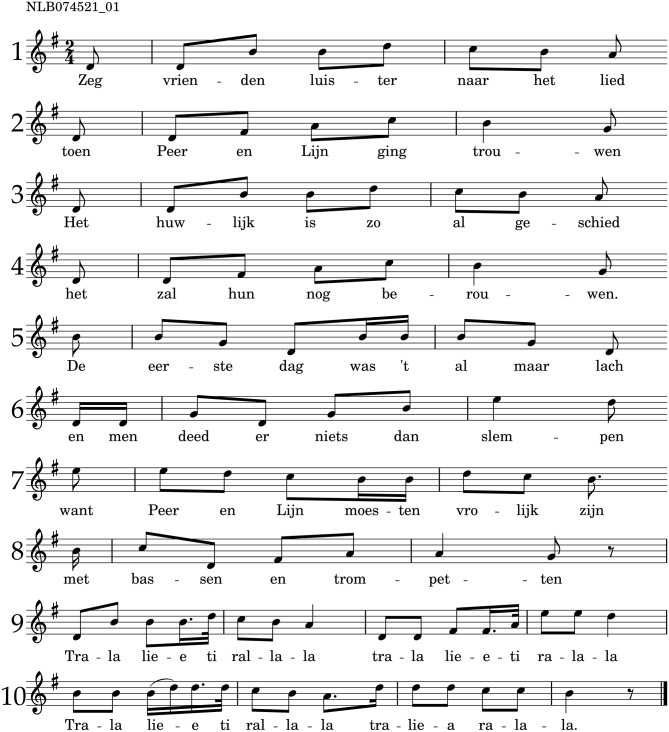
**An example melody from the test corpus, belonging to the tune family ***Van Peer en Lijn (1)***, which comprises six variants**. This melody is used to illustrate the formalizations of the hypotheses. The number on top of the sheet music shows the record number in the Dutch folk song database, the numbers left of the staves show the sequential phrase indices. A recording can be found at http://www.liederenbank.nl/sound.php?recordid=74521&lan=en.

This section describes the formalization of hypotheses on memorability of melodies^3^. For illustration purposes, we present a running example in Figure 1, a folk song melody from the tune family *Van Peer en Lijn (1)*, part of the test corpus. This melody has ten phrases and shows how under the current formalizations, different hypotheses arrive at different predictions of stability for each phrase. Throughout this section, we refer to a query phrase as *q*, which is taken from its source melody, *s*. The source melody's notes are referred to as *s*_*j*_. The query phrase starts at index *j* = *a* and has a length of *n* notes.

#### 2.2.1. Influence of phrase length

We test whether the length of the phrases has influence on their stability by defining the *phrase length* as the number of notes *n* of which a given phrase is composed.

(1) $$\begin{array}{r} Len\left(q\right)=n \end{array}$$

In the example melody, the shortest phrases (phrase 2 and 4) have a length of seven notes, the longest phrase (phrase 9) has 16 notes. According to the prediction of the list length effect, we would expect the second and fourth phrases to be more stable than the ninth phrase. Over the whole dataset, phrase length takes values in the range [3, 26] in the dataset, with a mean of $\bar{Len}=9.11$ and a standard deviation of **SD**(*Len*) = 2.23.

#### 2.2.2. Influence of rehearsal

Rehearsal is modeled based on phrase repetitions: if a phrase is repeated multiple times within a melody, it is subject to more rehearsal, hence it may be expected to be more stable. The resulting predictor, *phrase repetition*, is measured by counting the occurrences of a phrase in its source melody. All phrases in a melody *s* are defined as sets of notes *P*_*id*_. *id* refers to the sequential index of the phrase *P* in the melody. Each phrase's notes are represented by their onset from the start of the phrase and their pitch. The query phrase is a set of notes *Q* with the same representation. For every phrase *P*_*id*_ we determine its equality score to *Q* as follows:

(2) $$Eq(P_{id},Q)=\{\begin{array}{c} 1\\ 0 \end{array}\;\begin{array}{c} \;if\;P_{id}=Q\\ \text{otherwise} \end{array}$$

Then we measure the number of phrase repetitions *Rep* of the query phrase *q* by summing the equality scores of all *f* phrases *P*_*id*_ in the melody.

(3) $$\begin{array}{r} Rep\left(q\right)=\overset{f}{\underset{id=1}{\sum }}Eq\left(P_{id},Q\right) \end{array}$$

In the example melody, the first and second phrase repeat exactly as the third and fourth phrase, respectively. The other phrases do not repeat anywhere in the melody. This means that phrase repetition is *Rep* = 2 for the first four phrases, *Rep* = 1 for the other six phrases. This would lead to the prediction that the first four phrases are more stable than the last six phrases. Phrase repetition takes values in the range of [1, 4] in the dataset, with a mean of $\bar{Rep}=1.17$ and a standard deviation of **SD**(*Rep*) = 0.39.

#### 2.2.3. Influence of the primacy effect

We test the primacy effect based on the position of a phrase in its source melody. We formalize the *phrase position* as a given phrase's sequential index, *q*_*id*_, from *q*_*id*_ = 1 to *q*_*id*_ = *g* for all *g* phrases in the source melody. For the example melody of Figure 1, *g* = 10.

(4) $$\begin{array}{r} Pos\left(q\right)=q_{id} \end{array}$$

In the example melody, the first phrase has a value of *Pos* = 1, and the last phrase a value of *Pos* = 10. Phrase position takes values in the range of [1, 22] in the dataset, with a mean of $\bar{Pos}=3.44$ and a standard deviation of **SD**(*Pos*) = 2.06.

#### 2.2.4. Influence of expectancy

To quantify expectancy, we make use of two formalizations: one by Schellenberg (1997), which is based on observations from music theory, and one by Pearce and Wiggins (2004), which is based on statistical analysis of a background corpus.

We base both models on pitch intervals between consecutive notes. The pitch of a given note *pitch*(*s*_*j*_), or its height in the human hearing range, is represented by its MIDI note number. This entails that pitches are integers, in which a semitone difference between two pitches is indicated by a difference of one. The pitch interval between a note *s*_*j*_ and its predecessor *s*_*j*−1_ is defined by *pInt*(*s*_*j*_) = *pitch*(*s*_*j*_) − *pitch*(*s*_*j*−1_), where a positive sign indicates that the preceding note is lower, and a negative sign that the preceding note is higher. Both models make predictions for single *notes*, rather than whole phrases. We derive predictions for whole phrases through averaging the note values over the length of the phrase.

##### 2.2.4.1. Expectancy: music theory

The first component of Schellenberg's model, *pitch proximity*, states that listeners expect small steps between melody tones. The further a given note is away from its predecessor, the more unexpected it is. The model does not make any predictions for pitch intervals equal to or larger than an octave.

(5) $$PitchProx(s_{j})=\{\begin{array}{c} \left|pInt(s_{j})\right|\\ \text{undefined} \end{array}\begin{array}{c} \;if|\;pInt(s_{j})|\;<12\\ \text{otherwise} \end{array}$$

**Figure 2:**
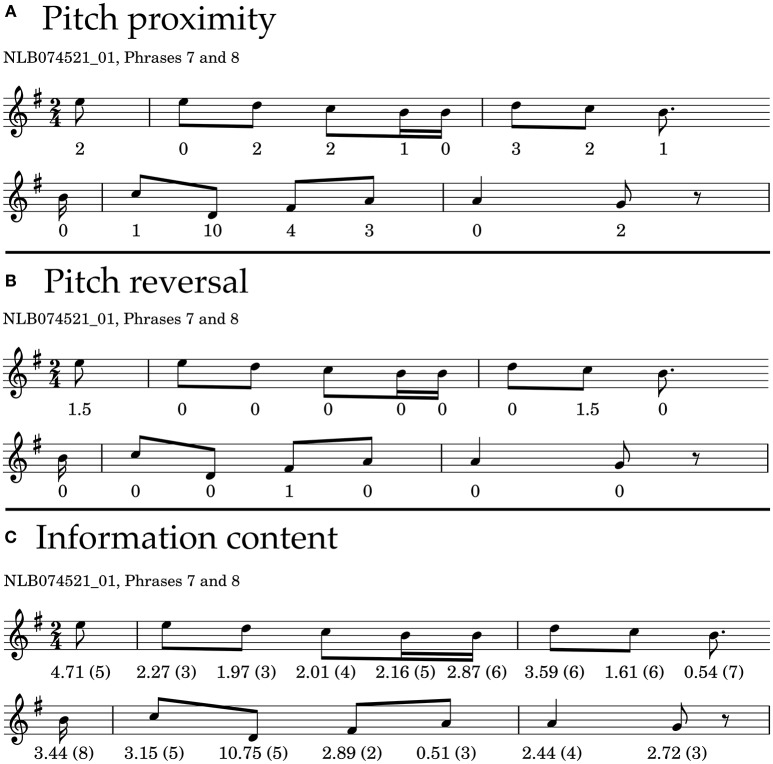
**Phrase 7 and 8 of the example melody, showing the values for each note resulting from different theories. (A)** Values according to Schellenberg's pitch-proximity principle. **(B)** Values according to Schellenberg's pitch-reversal principle. **(C)** Information Content, calculated with IDyOM, based on a background corpus. The numbers in brackets indicate how much context is considered to calculate information content, which in this case ranges from 2 (two previous notes considered) to 8 in the second phrase (eight previous notes considered).

In Figure 2A we show the first phrase of the example melody, with the pitch proximity values printed underneath each note, referring to the pitch interval to its preceding note. Note that the pitch interval, and therefore pitch proximity, is not defined for the first note of a melody, as there is no previous pitch from which a pitch interval could be measured.

To calculate the pitch proximity of a phrase, the pitch proximity values of the notes *s*_*j*_ belonging to a given phrase are averaged over the whole phrase, and the negative value of this average is used for easier interpretation, such that if a phrase has a high value of pitch proximity, its pitches are close to each other, while lower values indicate larger pitch intervals. Notes for which pitch proximity is not defined are discarded from the averaging procedure.

(6) $$\begin{array}{r} Prox\left(q\right)=-\frac{1}{n}\overset{a+n}{\underset{j=a}{\sum }}PitchProx\left(s_{j}\right) \end{array}$$

We show the pitch proximity values for the seventh and eighth phrase of the example melody in Figure 2A. The average proximity of the two phrases amounts to *Prox* = −13/9 = −1.44 and *Prox* = −20/7 = −2.85, respectively, which means that we would expect the seventh phrase to be more stable than the eighth phrase. Pitch proximity takes values in the range of [−6.0, 0.0] in the whole data set, with a mean of $\bar{Prox}=-2.01$ and a standard deviation of **SD**(*Prox*) = 0.69.

The other factor in Schellenberg's model is *pitch reversal*, which summarizes the long-standing observation from music theory that if leaps between melody notes do occur, they tend to be followed by stepwise motion in the opposite direction (Meyer, 1956). See the Supplementary Material for the quantification of this principle, which for a given melody note results in values ranging from *PitchRev*(*s*_*j*_) = −1, or least expected, to *PitchRev*(*s*_*j*_) = 2.5, or most expected. As with pitch proximity, we calculate the average reversal of a phrase through calculating the arithmetic mean of its constituent notes. As pitch reversal makes predictions based on two pitch intervals, it is not defined for the first two notes of a melody. Notes for which pitch reversal is not defined are discarded from the averaging procedure.

(7) $$\begin{array}{r} Rev\left(q\right)=\frac{1}{n}\overset{a+n}{\underset{j=a}{\sum }}PitchRev\left(s_{j}\right) \end{array}$$

We show the pitch reversal values for the seventh and eighth phrase of the example melody in Figure 2B. The average reversal of the two example phrases amounts to *Rev* = 3/9 = 0.33 and *Rev* = 1/7 = 0.14, respectively, which means that we would expect the seventh phrase to be more stable than the eighth phrase. Pitch reversal takes values in the range of [−0.5, 1.5] in the whole data set, with a mean of $\bar{Rev}=0.30$ and a standard deviation of **SD**(*Rev*) = 0.24.

##### 2.2.4.2. Expectancy: information theory

The IDyOM (Information Dynamics of Music) model by Pearce analyzes the frequencies of *n-grams* in a music collection, and based on these observed frequencies, assigns probabilities to notes in unseen melodies, given the notes preceding it. The preceding notes are also referred to as *context*. The length of the context can be set by the user. If the model cannot find a relevant *n-gram* of the context length specified by the user, it backtracks to shorter melodic contexts, and uses those frequencies to return the probability of a given note.

We let the model analyze our background corpus, with the melodies represented as pitch intervals. As we are interested in contexts of phrase length, we limit the *n-gram* length to the average phrase length of nine. We use IDyOM's long-term model, i.e., the model does not update itself while observing the query phrases, and we apply the interpolation weighting scheme *C*, which balances longer and shorter melodic contexts evenly. This was proven to be the best performing weighting scheme in experiments by Pearce (2005).

We express the expectancy of a given melodic segment through its average information content. Information content is the natural logarithm of the inverse probability ℙ(*s*_*j*_) of a note to occur given the previous melodic context, based on the probabilities of the background corpus. We choose information content rather than probability, as the logarithmic representation makes it possible to compare the typically small probability values in a more meaningful way. Information content is often also referred to as *Surprisal*, as its values increases as events get *less* expected.

We average the information content of all notes in a query phrase by their arithmetic mean, which is equivalent to a geometric mean of the probabilities. We call this average information content surprisal in the following, to indicate that higher values denote less expected phrases.

(8) $$\begin{array}{r} Sur\left(q\right)=\frac{1}{n}\overset{a+n}{\underset{j=a}{\sum }}\log\left(\frac{1}{\mathbb{P}\left(s_{j}\right)}\right) \end{array}$$

We show the information content for the seventh and eighth phrase of the example melody in Figure 2C. The context used to generate the information content is shown in brackets. The surprisal of the two example phrases amounts to *Sur* = 21.74/9 = 2.42 and *Sur* = 25.88/7 = 3.7, respectively, which means that we would expect the seventh phrase to be more stable than the eighth phrase. Surprisal takes values in the range of [1.15, 6.86] in the whole data set, with a mean of $\bar{Sur}=2.68$ and a standard deviation of **SD**(*Sur*) = 0.53.

#### 2.2.5. The influence of repeating motifs

As Müllensiefen and Halpern (2014) and Van Balen et al. (2015) found a relationship between repetitiveness of short motifs and the recall of a melody, we follow their procedure and use the FANTASTIC toolbox (Müllensiefen, 2009) to compute a frequency table of such short motifs *t* for each phrase. FANTASTIC uses a music representation which codes the relative pitches and durations of consecutive notes, see the Supplementary Material for a detailed description.

We follow Müllensiefen (2009) in their formalization to measure repeating motifs through entropy. The motifs are *n-grams* of character sequences representing the pitch and duration relationships between notes. The lengths of motifs to be investigated can be determined by the user. For each investigated motif length *l*, the frequency of unique motifs *v*_*z,l*_ is counted, and compared to the total number of motifs of that length *N*_*t,l*_ covering the phrase. The entropy *H*_*l*_ is then calculated from each unique motif's relative frequency *f*_*z,l*_, i.e., how often a given motif *v*_*z,l*_ occurs in a phrase with relation to all motifs of that length in the phrase.

The relative frequencies of all unique motifs are multiplied with their binary logarithm, summed, and divided by the binary logarithm of the number of all motifs of that length in the phrase (*N*_*u,l*_) for normalization. A value of *H* = 1.0 then indicates maximal entropy, and minimal repetitiveness: there are no repeating motifs of length *l* at all in the phrase; a lower value indicates that there are some repeating motifs.

(9) $$H(l)=-\frac{\sum_{z=1}^{N}t,lf_{z,l}\cdot \log_{2}f_{z,l}}{\log_{2}N_{u,l}}$$

The mean entropy of the motifs is then the average over all possible motif lengths. We analyze, in accordance with earlier work, motifs from two notes to six notes in length. We take the negative value of this average to define motif repetivity: the higher the average entropy, or the more distinct motifs in the phrase, the lower the repetivity.

(10) $$MR(q)=-\frac{\sum_{l=2}^{6}H(l)}{5}$$

To illustrate the concept, refer again to Figure 1, in which the second and fourth phrase, consist of repeated steps up by a third. This sequence can be subdivided into three identical sequences of two notes each (as the representation of the FANTASTIC toolbox does not distinguish between minor and major intervals): this would mean that this phrase has higher motif repetivity than, for instance, the sixth phrase. See the Supplementary Material for an example calculation of the motif repetivity of the second/fourth and the sixth phrase. The motif repetivity of the second/fourth phrase amounts to *MR* = −0.90, and of the sixth phrase to *MR* = −0.98, so we would expect the second and fourth phrase to be more stable than the sixth phrase. Motif repetivity takes values in the range of [−1.0, 0.0] in the whole data set, with a mean of $\bar{MR}=-0.92$ and a standard deviation of **SD**(*MR*) = 0.09.

### 2.3. Measuring statistical relationships

Since our outcome variables are binary, i.e., a given query phrase occurs or does not occur in a given melody, we model the statistical relationship between the likelihood that a given query phrase occurs and its properties through logistic regression. In logistic regression, the odds that an event happens are predicted as a function of one or multiple independent variables. The logarithm of the odds is also known as the *logit* function, where ℙ stands for the probability that an event happens:

(11) $$\begin{array}{r} logit\left(\mathbb{P}\right)=\log\left(\frac{\mathbb{P}}{1-\mathbb{P}}\right) \end{array}$$

The goal of logistic regression is to find a curve that best separates the true events from the false events. In our case, this means that we want to predict the probability ℙ that a given query phrase *q* has a match in a given melody *s*, based on the vector **F** of the independent variables hypothesized to contribute to long-term memorability of melodies.

(12) $$\begin{array}{r} logit\left(\mathbb{P}\right)=\beta \;\text{F}+\epsilon \end{array}$$

In this equation, β represents the slope of the prediction function, ϵ represents the random effects of the model, i.e., the random error for each melodic segment, assumed to be normally distributed. If the prediction of the probability of occurrence (i.e., the inverse logit of the prediction function) were perfect, this would lead to a curve separating the occurrences clearly from the non-occurrences.

However, the tune family dependent error of the computational method detecting occurrences needs to be taken into account. This could be done by separate logistic regression models for each tune family; yet this would mean that we could not globally estimate how well a specific hypothesis accounts for probability of occurrence. We therefore choose another solution to model the relationship between phrase properties and occurrence: a generalized linear mixed model (GLMM) which can model the variation of all data at the same time.

Generalized linear models are a framework in which relationships between independent variables and dependent variables of binomial, multinomial, ordinal and continuous distributions can be investigated. A special case of this framework are mixed models, in which not only a general random effect (ϵ), but also random effects for subgroups of the data can be taken into account. This way, we can model the tune family dependent error of the computational method. We assume that every tune family has a different intercept term in the model, i.e., the height at which the logistic regression curve crosses the *y* axis. Hence, the decision function between occurrence vs. non-occurrence of the model is shifted, depending on the tune family.

We again assume **F** as the vector representing the independent variables of the query phrases, β as the slope of the prediction function, ϵ as the random error, but now also take into account the random effect μ, based on the individual error of each tune family, summarized in the vector **tf**. Then the model can be formalized as follows:

(13) $$\begin{array}{r} logit\left(\mathbb{P}\right)=\beta \;\text{F}+\mu \;\text{t}\text{f}+\epsilon \end{array}$$

One could also think of the fixed effects, expressed by μ **tf** as the between-tune-family variance, and the random effects, expressed by ϵ, as the within-tune-family variance. Using this model, we test our hypotheses on possible correlates of long-term melody recall.

To be able to compare the independent variables derived from our hypotheses, we standardize all variables *x* of the predictor vector by subtracting the arithmetic mean $\bar{x}$, and dividing by the standard deviation **SD**(*x*) of a given variable.

(14) $$\begin{array}{r} {\text{F}}_{x}=\frac{x-\bar{x}}{\text{S}\text{D}\left(x\right)} \end{array}$$

This leads to the overall model for all phrase occurrences, in which units can be compared against each other. We apply a Generalized Linear Mixed Model with fixed slopes and random intercepts for each tune family through the R package LME4^4^ to the test corpus of the dataset containing 9,639 phrases from 147 tune families.

### 2.4. Model selection

We select the independent variables contributing to the strongest model predicting long-term memorability of folk song phrases, using the R library MuMIn^5^. This model selection compares all possible combinations of independent variables and ranks them based on their second-order Akaike information criterion (*AIC*_*c*_) (Hurvich and Tsai, 1989). The second-order Akaike information criterion penalizes the addition of extra parameters to a model, such that it strikes a balance between model fit and parsimony (Burnham and Anderson, 2004). Furthermore, we estimate the effect size of the best model with a technique to determine *R*^2^ of mixed models by Nakagawa and Schielzeth (2013).

## 3. Results

We show the best models selected from three degrees of freedom (3 df), with one model parameter, to nine degrees of freedom (9 df), with seven model parameters, in Table 1. The models' second-order Akaike information criteria decrease as more parameters get added, indicating better model fit. Our results show that the strongest model for the stability of melodic phrases is the full model with all independent variables: phrase length, phrase repetition, phrase position, pitch proximity, pitch reversal, surprisal and motif repetivity. This model yields an *AIC*_*c*_ lower by 70.65 than the second best model. Table 2 shows the estimated prediction coefficients, the variances of the tune family dependent error and the residual error for the full model, as well as the model fit in *R*^2^. The fixed effects alone, marginalized, explain $R_{marginal}^{2}=0.05$, or about 5% of the variance, which is a mid-sized effect for mixed models (Cohen, 1992; Kirk, 1996). When the tune family dependent random effects are considered along with the fixed effects ($R_{conditional}^{2}$), 22% of the variation in the data is explained.

**Table 1:** **The best models for different degrees of freedom, from 3 df with one parameter, to 9 df with seven parameters**.

**Parameter estimate**	**3 df**	**4 df**	**5 df**	**6 df**	**7 df**	**8 df**	**9 df**
Surprisal	−0.27	−0.29	−0.30	−0.30	−0.29	−0.24	−0.24
Phrase length		−0.32	−0.32	−0.33	−0.30	−0.31	−0.30
Phrase position			−0.10	−0.12	−0.12	−0.10	−0.10
Phrase repetition				0.08	0.09	0.09	0.09
Motif repetivity					0.08	0.08	0.08
Average proximity						0.09	0.10
Average reversal							0.05
*AIC_c_*	209159.8	206889.7	206584.4	206355.5	206157.6	206012.1	205941.5

*For each model, the second order Akaike information criterion (AIC_c_) is shown, with lower values indicating better model fit. Surprisal is the parameter which leads to the best model with only one predictor; the other parameters are listed in the order by which they are added, leading to the best model fit when all parameters are used*.

**Table 2:** **The parameters of the best model of the model selection: estimated regression coefficient $\hat{\beta }$ and 95% confidence interval for ***phrase length, phrase repetitions*** within the source melody, ***phrase position*** in the source melody, ***pitch proximity*** and ***pitch reversal*** as defined by Schellenberg (1997), ***expectancy***, as defined by IDyOM (Pearce and Wiggins, 2004), and ***motif repetivity***, as defined by Müllensiefen (2009)**.

**Parameter**	**$\hat{\beta }$**	**95% CI**
Intercept	−0.22	[−0.35, −0.08]
Surprisal	−0.24	[−0.25, −0.22]
Phrase length	−0.30	[−0.32, −0.29]
Phrase position	−0.10	[−0.11, −0.09]
Phrase repetition	0.09	[0.08, 0.10]
Motif repetivity	0.08	[0.07, 0.09]
Average proximity	0.10	[0.08, 0.11]
Average reversal	0.05	[0.04, 0.06]
σ_*tf*_	0.84	[0.74, 0.95]
$R_{marginal}^{2}$		0.05
$R_{conditional}^{2}$		0.22

*At the bottom of the table we report the standard deviation of the random effect (tune family), as well as the marginalized and conditional R^2^ calculated according to Nakagawa and Schielzeth (2013)*.

The prediction coefficients show that phrase length and surprisal possess most predictive power: with increase of a given query phrase's length, its stability decreases. Higher expectancy leads to increased stability. Furthermore, the coefficients also indicate that earlier phrases tend to be more stable, as with an increase in the phrase index, the odds that a query phrase occurs in a given melody are decreased. Moreover, an increase in pitch proximity, or a decrease in the average size of the pitch intervals in a phrase, leads to a higher chance of an occurrence. More repetitions of a query phrase also result in the increased odds of occurrence. Pitch reversal and motif repetivity contribute least strongly to the model, but the signs of the parameters are as expected: if a phrase confirms expectations of pitch reversal, its odds of occurrence are increased, and likewise, if a phrase contains many repeating motifs, its odds of occurrence are increased.

We also tested the model for multicollinearity, confirming that the approximate correlations of parameter estimates do not exceed 0.6, which justifies our treatment of the model parameters as independent predictors.

**Figure 3:**
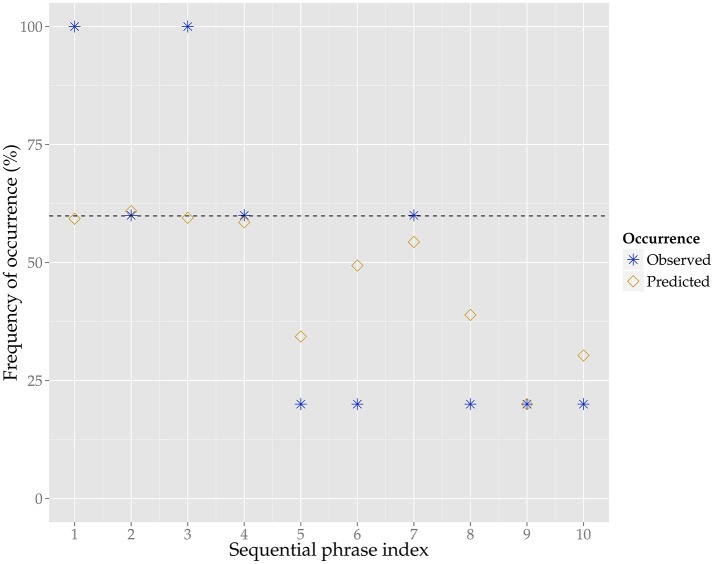
**The predicted (yellow diamonds) and observed (blue stars) frequency of occurrence, in percent, for the ten phrases of the example melody**. The predictions are generated by the generalized linear mixed model, for the model parameters see Table 2. The observed frequency of occurrence is based on how many of the five variants, other than the example melody, contain a given phrase from the example melody. The dashed line shows the model's intercept for frequency of occurrence for this tune family, which is at 58%, meaning that is slightly more likely for the phrases of this tune family to occur in the respective variants than not.

To illustrate the predictions of the model, we show the predicted as well as the observed frequency of occurrence for the ten phrases of the example melody in Figure 3. According to the model, the first four phrases have the highest probability of occurrence, and indeed these phrases also have the highest observed frequency of occurrence (i.e., stability). The predictions do differ from many of the observed values, as for instance the higher stability of phrase 1 and 3 as compared to phrase 2 and 4 is not captured by the model.

## 4. Discussion

The current research shows that folk song collections are a valuable resource for studying the relationship between melody variation and memorability. All proposed hypotheses relating to recall in general and music recall in particular contribute to prediction of folk song variation, as model selection among all combinations of parameters leads to a model with all hypotheses as predictors.

Of course, the variation that is explained with the current model is still rather low at *R*^2^ = 0.05. This might mean that there are potentially more, and stronger predictors for melody variation that have not been tested in this study. It is also good to keep in mind that the phrase occurrences in folk songs do not represent “clean” experimental data in which all aspects but melody recall are controlled. The ecological validity comes at the cost of potential noise. Some aspects that might deteriorate the observed variation are (a) the computational method to detect occurrences; (b) the inherent ambiguity of phrase occurrences, i.e., humans do not agree on occurrences perfectly (Janssen et al., in press); (c) a bias in the corpus toward specific regions and demographic groups (Grijp, 2008).

Alternatively, one could assume that a large proportion of melody variation is a result of drift, and therefore random (Henrich and Boyd, 2002). Therefore, it is enlightening that the hypotheses *do* contribute to explaining variation in the dataset, in spite of potential noise in the data. Memorability predicts the amount of melodic variation, or stability, as follows: phrases which resist change should be short (list length effect, hypothesis 1) and contain little surprising melodic material (i.e., low surprisal, a formalization of expectancy, hypothesis 4a). Moreover, it is beneficial if a phrase occurs relatively early in a melody (primacy effect, hypothesis 3), and has mostly small pitch intervals (i.e., high average proximity, a formalization of expectancy, hypothesis 4a). The repetition of a phrase in its source melody also contributes to its memorability (rehearsal effect, hypothesis 2), even though this effect is somewhat weaker in our analysis than other predictors. Average reversal, or the tendency for a melody to adhere to the gap fill principle, i.e., following a leap by stepwise motion in the opposite direction (expectancy, hypothesis 4a) and motif repetivity within the phrase (hypothesis 5) seem to account for long-term memorability to a more limited extent. All predictors related to expectancy indicate that more expected melodic material increases stability, leading us to reject hypothesis 4b.

As for possible drawbacks of the presented study, the three predictors related to expectancy (average proximity, average reversal and surprisal) share the disadvantage that for the first few notes of a melody, no or little information on expectancy is available. This means that there is a potential imbalance between the initial and later phrases of a melody, as the predictor values of initial phrases are based on less information. The alternative, treating every phrase as isolated, so that no context from previous phrases is used for creating expectancy values, seemed unrealistic, however, as the recall of phrases is cued by previous melodic material (cf. Rubin, 1995, p. 190). For the current folk song collection, in which the same melody is sung multiple times with different verses, it may be interesting to investigate in how far considering the end of a given melody as the melodic context for the start of this melody influences expectancy predictions.

The expectancy predictors defined by Schellenberg (1997), average proximity and average reversal, may be comparatively unsuccessful model parameters as they were not necessarily designed to be averaged for a longer melodic context: they were defined to quantify the fulfillment of listener expectations at a given note. However, these predictors still contribute to a better model, which shows that they capture some information on memorability which may predict variation of melodies in this corpus.

The relatively low contribution of motif repetivity as a predictor for melodic variation may be partly ascribed to the fact that the phrases are very short melodic material, and as such rarely contain repeated motifs. It would be interesting to investigate if motif repetivity increases stability of longer melodic contexts, e.g., full folk song melodies. For the current analysis of phrases with an average length of nine notes, which are unlikely to contain repeated motifs longer than four notes, it may be sufficient to limit the maximal *n-gram* length to four notes for future research on motif repetivity in phrases. To hold our use of the method comparable to earlier research, we decided to analyze motifs of the same lengths as previous authors. Moreover, there is no disadvantage to considering longer *n-grams* other than longer computation time, as the FANTASTIC toolbox automatically disregards *n-grams* which are longer than the length of a phrase.

With the current approach, we cannot address the influence of other memory effects on melody variation, such as fill-in effects, spacing effects or confusion errors. Fill-in effects (Conrad and Hull, 1964), which lead to the later inclusion of an item that was skipped earlier in serial recall, may also play a role in melody recall. This might be observed, for instance, if melodic material within a phrase or melody is rearranged, such that a motif which usually starts a melody appears later instead. With the current method, these effects would be missed, as only the amount of melodic variation, but not the kind of melodic variation, is investigated. In the same vein, the spacing effect from free recall (c.f. Hintzman, 1969; Madigan, 1969), which relates to the space between rehearsals of items, cannot be studied on the basis of phrases, which do not necessarily repeat within a melody, and if they do, usually are not spaced far apart. Instead, shorter melodic contexts might be interesting to study to this effect.

Furthermore, confusion errors (Page and Norris, 1998), which in serial recall of words lead to the erroneous recall of acoustically similar words, might also be interesting to study for melody variation. This might occur if instead of a melodic phrase in a given folk song, a similar phrase from another folk song is recalled. As our study analyzes variation per tune family and not across different tune families, melodic material that might correspond between different folk songs is not identified as such.

As our analysis of an existing folk song corpus highlighted some mechanisms behind melodic variation which may be tied to memorability of melodies, this shows that it would certainly be fruitful to perform more studies based on computational music analysis: such research could be performed on the present folk song corpus to investigate other potential effects of recall (cf. Olthof et al., 2015), or our methods could be applied to other music collections, to see whether our findings can be replicated with respect to melodic variation in other musical traditions.

Next to further computational studies, it would certainly also be an important future contribution to test the predictions on melodic variation in an experiment with human participants. Could the amount of variation when melodies are learned in an experimental setting also be predicted through important parameters of our corpus analysis, e.g., through surprisal, phrase length and phrase position?

As the melodies in the Meertens Tune Collections were recorded or notated long after the singers or editors had learned the melodies, it would also be interesting to investigate whether immediate recall of melodies in a laboratory setting leads to different kinds of variation than if melodies are recalled weeks or months later. As such, the present collection, and other folk song collections, might be an overlooked resource to study recall and long term memory for melodies.

## Author contributions

BJ performed the analyses of musical and statistical data and wrote the manuscript. JB advised on the statistical analysis and edited the manuscript. HH advised on the analysis of musical data and edited the manuscript.

## Funding

BJ is supported by the Computational Humanities Programme of the Royal Netherlands Academy of Arts and Sciences, under the auspices of the Tunes & Tales project. For further information, see http://ehumanities.nl. JB is supported by the Amsterdam Brain and Cognition Talent grant. HH is supported by a Horizon grant (317-70-10) of the Netherlands Organization for Scientific Research (NWO).

## Acknowledgments

We thank our colleagues from the Music Cognition Group for feedback at various stages of the research, Andrei Teodorescu and Esther Adi-Japha for their invaluable comments on the manuscript, the folk song experts at the Meertens Institute for their annotations and data curation, and Peter van Kranenburg for advice and feedback on the current research, as well as the publication of the Meertens Tune Collections.

### Conflict of interest statement

The authors declare that the research was conducted in the absence of any commercial or financial relationships that could be construed as a potential conflict of interest.

## Footnotes

^1^https://code.soundsoftware.ac.uk/projects/idyom-project.

^2^www.liederenbank.nl/mtc.

^3^The implementations of the hypotheses can be found at https://github.com/BeritJanssen/Stability.

^4^https://CRAN.R-project.org/package=lme4.

^5^https://CRAN.R-project.org/package=MuMIn.
