# Supplementary material for: Predicting Variation of Folk Songs: A Corpus Analysis Study on the Memorability of Melodies
Source: Front Psychol. 2017 Apr 25;8:621. doi: 10.3389/fpsyg.2017.00621 (PMC5403935; doi:10.3389/fpsyg.2017.00621)
Supplement: Supplementary file 1 [file Presentation1.pdf]

Supplementary Material: Memorability of folk songs: evidence from corpus analysis

Berit Janssen*, John Ashley Burgoyne and Henkjan Honing
*Correspondence:
Berit Janssen:
berit.janssen@meertens.knaw.nl

1 DETAILS ON DETECTING PHRASE OCCURRENCES

We use a combination of three pattern matching methods, which have been shown to agree best with human judgements of phrase occurrences (Janssen et al., Under Revision): city-block distance (Steinbeck, 1982), local alignment (Smith and Waterman, 1981) and structure induction (Meredith, 2006).

1.1 Music representations

For city-block distance and local alignment, melodies are represented as pitch sequences. Pitches (the heights of the melody notes in the human hearing range), are represented by integers, derived from their MIDI note numbers. The notes in the pitch sequences were weighted by their duration, i.e., a given pitch is repeated depending on the length of the notes. We represent a crotchet or quarter note by 16 pitch values, a quaver or eighth note by 8 pitch values, and so on. Note onsets of small duration units, especially triplets, may fall between these sampling points, which shifts their onset slightly in the representation. Structure induction uses *(onset, pitch)* pairs to represent notes in the melodies.

In order to deal with transposition differences in folk songs, Van Kranenburg et al. (2013) transpose melodies to the same key using pitch histogram intersection. We take a similar approach. For each melody, a pitch histogram is computed with MIDI note numbers as bins, with the count of each note number weighted by its total duration in a melody. The pitch histogram intersection of two histograms h_s and h_t, with shift σ is defined as

$$PHI(h_s, h_t, \sigma) = \sum_{k=1}^{r} \min(h_{s,k+\sigma}, h_{t,k}), \quad \text{(S1)}$$

where k denotes the index of the bin, and r the total number of bins. We define a non-existing bin to have value zero. For each tune family, we randomly pick one melody and for each other melody in the tune family we compute the σ that yields a maximum value for the histogram intersection, and transpose that melody by σ semitones. This process results in *pitch-adjusted sequences*.

To deal with different notations for the durations of notes, we perform a similar correction for the durations of notes. Analogous to Equation S1, we define a duration histogram intersection of two duration histograms h_t and h_s, of which the σ minimizing DHI will be chosen as the designated shift.

$$DHI(h_t, h_s, \sigma) = \sum_{k=1}^{r} \min(h_{t,k+\sigma}, h_{s,k}), \quad \text{(S2)}$$

This σ is then used to calculate the multiplier of the onsets of melody t with relation to melody s, before transforming the pitch and duration values of melody t into a duration weighted pitch sequence:

$$Mult(t, s) = 2^{\sigma} \tag{S3}$$

1.2 City-block distance

For city-block distance, the query sequence q, with pitch values q_i, is compared with every sequence p of the same length, with pitch values p_i, from the melody being searched for matches. If many pitch values are identical, city-block distance is small.

$$dist(q, p) = \sum_{i=1}^{n} |q_i - p_i| \tag{S4}$$

From each melody, we choose the pitch sequences p which have the lowest city-block distance to the query sequence, and determine their position in the melody.

1.3 Local alignment

To compute the optimal local alignment, a matrix A is recursively filled according to equation S5. The matrix is initialized as $A(i, 0) = 0, i \in \{0, \ldots, n\}$, and $A(0, j) = 0, j \in \{0, \ldots, m\}$. $W_{insertion}$ and $W_{deletion}$ define the weights for inserting an element from melody s into segment q, and for deleting an element from segment q, respectively. $subs(q_i, s_j)$ is the substitution function, which gives a weight depending on the similarity of the notes q_i and s_j.

$$A(i, j) = \max \begin{cases} A(i-1, j-1) + subs(q_i, s_j) \\ A(i, j-1) + W_{insertion} \\ A(i-1, j) + W_{deletion} \\ 0 \end{cases} \tag{S5}$$

We apply local alignment to pitch adjusted sequences. In this representation, local alignment is not affected by transposition differences, and it should be robust with respect to time dilation. For the insertion and deletion weights, we use $W_{insertion} = W_{deletion} = -0.5$, and we define the substitution score as

$$subs(q_i, s_j) = \begin{cases} 1 & \text{if} \quad q_i = s_j \\ -1 & \text{otherwise} \end{cases} . \tag{S6}$$

We normalize the maximal alignment score by the number of notes n in the query segment to receive the similarity of the found match with the query segment. The position of the pitch sequence associated with the maximal alignment score is determined through backtracing.

$$sim(q, s) = \frac{1}{n} \max_{i,j}(A(i, j)) \tag{S7}$$

1.4 Structure induction

Structure induction measures the difference between melodic segments through so-called translation vectors. The translation vector $\mathbf{T}$ between points in two melodic segments can be seen as the difference between the points q_i and s_j in onset, pitch space.

$$\mathbf{T} = \begin{pmatrix} q_{i,onset} \\ q_{i,pitch} \end{pmatrix} - \begin{pmatrix} s_{j,onset} \\ s_{j,pitch} \end{pmatrix} \quad \text{(S8)}$$

The maximally translatable pattern (MTP) of a translation vector $\mathbf{T}$ for two melodies q and s is then defined as the set of melody points q_i which can be transformed to melody points s_j with the translation vector $\mathbf{T}$.

$$MTP(q, s, \mathbf{T}) = \{q_i | q_i \in q \wedge q_i + \mathbf{T} \in s\} \quad \text{(S9)}$$

We use the pattern matching method SIAM, defining the similarity of two melodies as the largest set match achievable through translation with any vector, normalized by the length n of the query melody:

$$sim(q, s) = \frac{1}{n} \max_{\mathbf{T}} |MTP(q, s, \mathbf{T})| \quad \text{(S10)}$$

The maximally translatable patterns leading to highest similarity are selected as matches, and their positions are determined through checking the onsets of the first and last note of the MTPs.

1.5 Combination of the measures

The similarity thresholds which result in the best agreement between human annotations of phrase occurrences and algorithmically determined matches were found through optimization on the training corpus. For a given query phrase, all similarity measures were used to determine whether or not a match was found in a given melody. For city-block distance, matches with $dist(q, p) \leq 0.9792$, for local alignment, matches with $sim(q, s) \geq 0.5508$, and for structure induction, matches with $sim(q, s) \geq 0.5833$ are retained. Only if at least two of the three similarity measures retained matches, the melody in question was considered to contain an occurrence.

2 DETAILS ON THE FORMALIZATION OF HYPOTHESES

2.1 Pitch reversal

Pitch-reversal is the linear combination of two other principles, registral direction and registral return. The principle of registral direction states that after large implicative intervals, a change of direction is more expected than a continuation of the direction. The tritone, or six semitones, is not defined in this principle.

$$\text{PitchRev}_{dir}(s_j) = \begin{cases} 0 \quad \text{if} \quad \text{pInt}(s_{j-1}) < 6 \\ 1 \quad \text{if} \quad 6 < \text{pInt}(s_{j-1}) < 12 \quad \text{and} \quad \text{pInt}(s_j) \cdot \text{pInt}(s_{j-1}) < 0 \\ -1 \quad \text{if} \quad 6 < \text{pInt}(s_{j-1}) < 12 \quad \text{and} \quad \text{pInt}(s_j) \cdot \text{pInt}(s_{j-1}) > 0 \\ \text{undefined} \quad \text{otherwise} \end{cases} \quad \text{(S11)}$$

The other component of pitch-reversal, registral return, states that if the realized interval has a different direction than the implicative interval, the size of the intervals is expected to be similar, i.e. they should not differ by more than two semitones. If the implicative interval describes a tone repetition, or if the difference between two consecutive pitch intervals of opposite direction is too large, pitch-reversal is zero, otherwise it has the value of 1.5.

$$\text{PitchRev}_{ret}(s_j) = \begin{cases} 1.5 \quad \text{if} \quad \text{pInt}(s_j) > 0 \quad \text{and} \quad \text{pInt}(s_{j-1}) + \text{pInt}(s_j) <= 2 \\ 0 \quad \text{otherwise} \end{cases} \tag{S12}$$

Combined, registral direction and registral return form the pitch-reversal principle.

$$\text{PitchRev}(s_j) = \text{PitchRev}_{dir}(s_j) + \text{PitchRev}_{ret}(s_j) \tag{S13}$$

Figure S1, drawn after a figure by Schellenberg, shows a schematic overview for the different values pitch reversal can take under different conditions.

2.2 Motif repetivity

In Figure S2 we show the second, also fourth, and sixth phrase of the example melody. FANTASTIC (Müllensiefen, 2009) represents relationship between adjacent notes as follows: pitches can either stay the same (s1), move up or down by a diatonic pitch interval (e.g., u4, d2). In this representation, it does not matter whether, e.g, a step down (d2) contains one or two semitones. Durations either stay equal (e), get quicker (q) or longer (l).

We will refer to the string representations of the motifs, in accordance with Müllensiefen and Halpern (2014), as *M-Types*. The second/fourth phrase of the example melody consists of six M-types, of which one repeats three times, so there are four unique M-types. This leads to the entropy of two-note M-Types:

$$H(2) = \frac{3/6 \log_2 3/6 + 3 \cdot (1/6 \log_2 1/6)}{\log_2 1/6} = \frac{-0.5 - 3 \cdot 0.43}{-2.58} = 0.69 \tag{S14}$$

These M-types can be combined to the following three-note M-Types:

s1q_u3e
u3e_u3e
u3e_d2l
d2l_d3q

One M-Type, "u3e_u3e", is repeated. In total, there are five M-Types in the melody, and four unique M-Types. This means that the entropy for length $l = 3$ for this phrase is

$$H(3) = \frac{3/5 \log_2 3/5 + 3 \cdot (1/5 \log_2 1/5)}{\log_2 1/5} = \frac{-0.44 - 3 \cdot 0.46}{-2.32} = 0.79 \tag{S15}$$

There are no repeated four-note M-Types, so the entropy of M-Types of length $l = 4$ for this phrase is maximal, $H(4) = 1.0$. This means that all longer M-types also have maximal entropy, and the motif

repetivity, the negative average of the entropies for all lengths of *mtypes*, is $MR = -(0.69 + 0.79 + 3 \cdot 1.0)\ /\ 5 = -0.90$.

The sixth phrase of the example melody (the second phrase shown in the figure) consists of seven M-Types, of which only one (u4l) appears twice. This leads to the entropy of two-note M-Types:

$$H(2) = \frac{2/7 \log_2 2/7 + 5 \cdot (1/7 \log_2 1/7)}{\log_2 1/7} = \frac{-0.52 - 5 \cdot 0.40}{-2.81} = 0.89 \quad \text{(S16)}$$

For the longer M-Types, there are no repetitions, hence the entropy is maximal at $H(3, 4, 5, 6) = 1.0$. This leads to a motif repetivity of $MR = -(0.89 + 4 \cdot 1.0\ /\ 5) = -0.98$.

REFERENCES

Janssen, B., van Kranenburg, P., and Volk, A. (Under Revision). Finding Occurrences of Melodic Segments in Folk Songs: a Comparison of Symbolic Similarity Measures. *Journal of New Music Research*

Van Kranenburg, P., Volk, A., and Wiering, F. (2013). A Comparison between Global and Local Features for Computational Classification of Folk Song Melodies. *Journal of New Music Research* 42, 1–18. doi:10.1080/09298215.2012.718790

Meredith, D. (2006). Point-set algorithms for pattern discovery and pattern matching in music. In *Content-Based Retrieval. Dagstuhl Seminar Proceedings 06171*, eds. T. Crawford and R. C. Veltkamp (Dagstuhl, Germany)

Müllensiefen, D. (2009). *FANTASTIC: Feature ANalysis Technology Accessing STatistics (In a Corpus): Technical Report*. Tech. rep., Goldsmiths University of London, UK

Müllensiefen, D. and Halpern, A. R. (2014). The Role of Features and Context in Recognition of Novel Melodies. *Music Perception* 31, 418–435

Schellenberg, E. G. (1997). Simplifying the Implication-Realization Melodic Expectancy. *Music Perception: An Interdisciplinary Journal* 14, 295–318

Smith, T. and Waterman, M. (1981). Identification of common molecular subsequences. *Journal of Molecular Biology* 147, 195–197. doi:10.1016/0022-2836(81)90087-5

Steinbeck, W. (1982). *Struktur und Ähnlichkeit. Methoden automatisierter Melodienanalyse* (Kassel: Bärenreiter)

3 SUPPLEMENTARY FIGURES

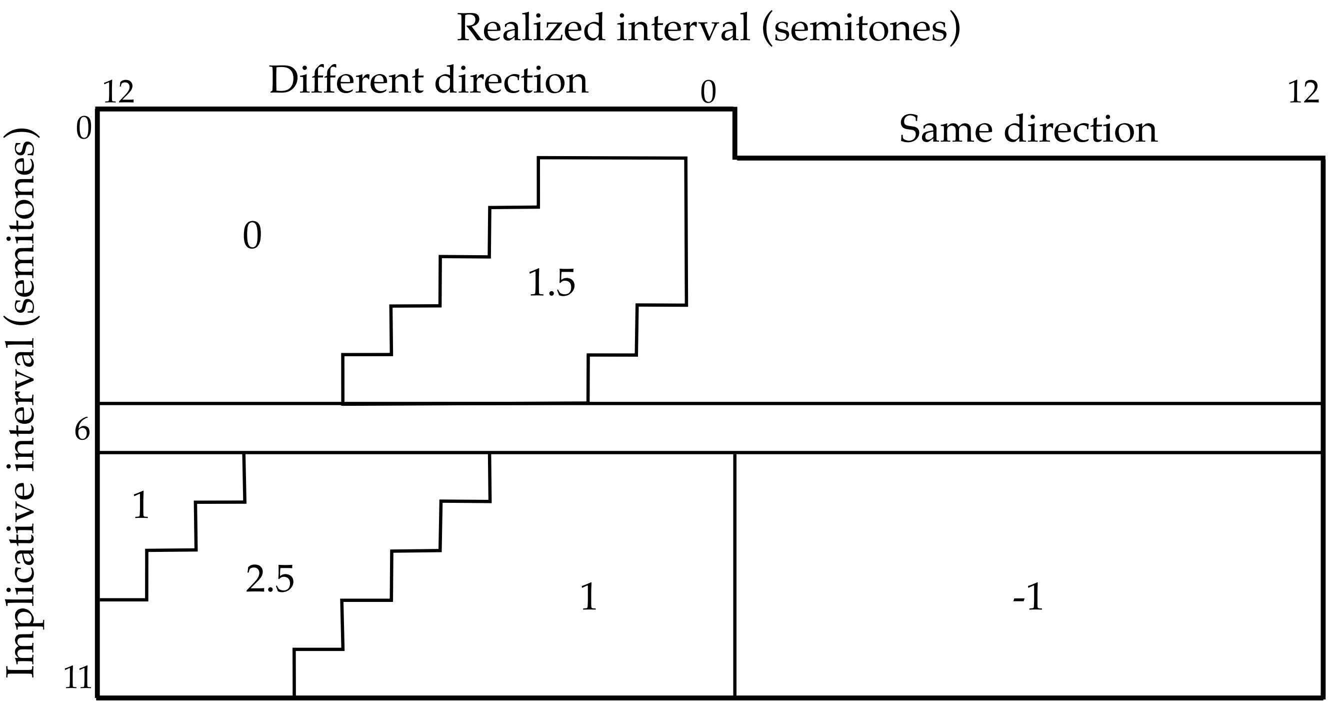

Figure S1. A visualization of the pitch reversal principle as defined by Schellenberg (1997), drawn after his figure. The vertical axis represents the size of the implicative interval, from 0 to 11, and the horizontal axis the size of the realized interval, from 0 to 12, which can have either the same direction (right side of the panel) or a different direction (left side of the panel).

NLB074521_01, Phrases 2/4 and 6

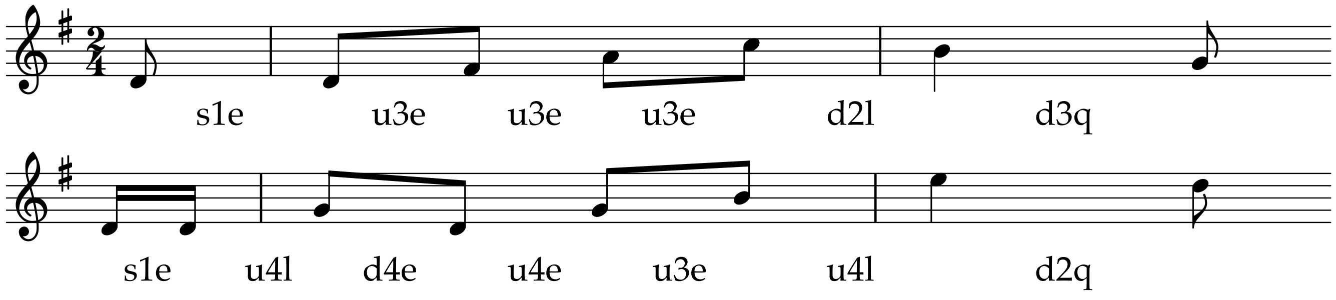

Figure S2. The second, also fourth, and sixth phrase of the example melody with symbols representing the pitch and duration relationships between adjacent notes. Notes can either stay at the same pitch (s1), or move up or down in a diatonic pitch interval (e.g., u4, d2). Durations can either be equal (e), quicker (q), or longer (l).
